# Postural sway serves as a predictive biomarker in balance and gait assessments for diabetic peripheral neuropathy screening: a community-based study

**DOI:** 10.1186/s12984-025-01644-6

**Published:** 2025-06-02

**Authors:** Yun-Ru Lai, Wen-Chan Chiu, Chi-Ping Ting, Yi-Fang Chiang, Ting-Yin Lin, Hui-Ching Chiang, Chih-Cheng Huang, Cheng-Hsien Lu

**Affiliations:** 1 Department of Neurology, Kaohsiung Chang Gung Memorial Hospital, Chang Gung University College of Medicine, No. 123, Ta Pei Road, Niao Sung District, Kaohsiung, 833 Taiwan; 2 Department of Hyperbaric Oxygen Therapy Center, Kaohsiung Chang Gung Memorial Hospital, Chang Gung University College of Medicine, Kaohsiung, Taiwan; 3 Department of Internal Medicine, Kaohsiung Chang Gung Memorial Hospital, Chang Gung University College of Medicine, Kaohsiung, Taiwan; 4 Department of Nursing, Kaohsiung Chang Gung Memorial Hospital, Chang Gung University College of Medicine, Kaohsiung, Taiwan; 5 Department of Neurology, Chi-Mei Medical Center, Tainan, Taiwan; 6 Department of Biological Science, National Sun Yat-Sen University, Kaohsiung, Taiwan; 7 Doctoral Program of Clinical and Experimental Medicine, College of Medicine, National Sun Yat-Sen University, Kaohsiung, Taiwan; 8 Department of Neurology, Xiamen Chang Gung Memorial Hospital, Xiamen, Fujian China

**Keywords:** Balance and gait assessments, Diabetic sensorimotor polyneuropathy, Diabetic peripheral neuropathy, Large- and small-fiber dysfunction, Nerve conduction studies, Postural sway velocity, Sudoscan, Toronto clinical neuropathy score

## Abstract

**Background:**

Traditional screening methods for diabetic peripheral neuropathy (DPN) can be time-consuming in community settings. Balance and gait impairments are common in individuals with DPN, but these functional impairments are often not detectable with standard neurological examinations. This study aimed to examine whether quantitative balance and gait assessment could serve as a viable alternative screening tool for DPN.

**Methods:**

All participants were recruited from a community-based daycare center and underwent peripheral nerve function assessments, including the Toronto Clinical Neuropathy Score (TCNS), sural nerve conduction studies (amplitude and velocity) for large fiber function, and Sudoscan testing for small fiber function. Subsequently, participants underwent balance and gait assessments, including static postural sway measurements and gait analysis of spatiotemporal parameters and joint range of motion (ROM) assessment during walking.

**Results:**

Of the 146 participants, 35 had diabetes, including 22 with DPN, while 111 were healthy controls. Participants with DPN demonstrate increased postural sway velocity and total path length, along with reduced gait speed, shorter stride length, and decreased range of motion in hip flexion and extension. The logistic regression analysis identified diabetes duration and postural sway velocity as the only significant predictors of DPN presence. Postural sway velocity demonstrated strong correlations with elevated TCNS, reduced sural sensory nerve action potential and sensory nerve conduction velocity, and lower Sudoscan values in hands and feet. Additionally, receiver operating characteristic analysis yielded a sensitivity of 68.2%, specificity of 85.5%, and an area under the curve of 0.76, with a cut-off value of 0.98 cm/s.

**Conclusions:**

Balance and gait impairments are prevalent among participants with DPN. This study supports the integration of balance and gait assessments into community-based screening protocols to facilitate early identification and intervention. Postural sway velocity emerged as a practical early biomarker for the screening of DPN.

## Introduction

Diabetic sensorimotor polyneuropathy (DSPN), the most common form of diabetic peripheral neuropathy (DPN), is characterized by symmetric distal degeneration of peripheral nerves and impaired nerve regeneration [1]. DPN involves dysfunction of both large and small nerve fibers, affecting intrinsic foot muscles, tactile sensitivity, vibration perception, plantar skin integrity, and proprioceptive feedback. These deficits contribute to altered gait patterns and impaired balance [2–5].

Large myelinated fibers transmit essential proprioceptive and vibratory signals from the lower limbs. In DPN, progressive dysfunction of these fibers diminishes the accuracy and speed of proprioceptive input, compromising the detection of ground reaction forces and joint positions in real-time. This sensory deficit often presents as impaired balance responses, prolonged double-support phases, and an increased risk of falls [6].

Small unmyelinated fibers, particularly sympathetic fibers, regulate sweat gland function and skin temperature, playing a key role in maintaining thermal homeostasis. Damage to these fibers leads to impaired moisture control and reduced thermoregulation. During walking, heat generated by the body is normally dissipated through sweat secretion. When sweat gland function is impaired, the ability to regulate body temperature is diminished, potentially leading to overheating, which can adversely affect balance and gait [7]. While their direct role in dynamic balance remains less well defined, small fiber dysfunction has been associated with sudomotor impairment and may signal early peripheral nerve involvement.

Moreover, small fiber abnormalities often precede large fiber damage [8]. These early-phase autonomic impairments may subtly increase the risk of balance deficits and gait instability before more overt neuropathic symptoms become clinically apparent. Early prediction of DPN is essential for identifying at-risk individuals [9]. The American Diabetes Association guideline recommends a careful history and a combination of at least two assessments—one targeting small fibers and one targeting large fibers—for effective screening and diagnosis of DSPN in routine clinical practice [10]. In the general health care system, combined large- and small-fiber electrophysiological testing, including validated nerve conduction studies (NCS) and/or Sudoscan™ remains necessary [11, 12].

Postural sway is a kinematic parameter that quantifies the displacement of the body’s center of pressure (COP) or center of mass over time during static or dynamic stance, reflecting the body’s ability to maintain balance [13]. Increased sway velocity is often indicative of poorer postural stability. Postural sway is typically quantified using force-plate technology or wearable sensor systems [14–16], which track parameters including sway area (cm²), length (cm), velocity (cm/s), and excursion in both the anteroposterior (AP) and mediolateral (ML) directions [6]. Quantitative evidence suggests that postural sway is increased in individuals with diabetes, even under eyes-open conditions. This degradation in sensory feedback, particularly from the plantar surface, may impair the ability to make rapid and accurate postural adjustments, resulting in increased sway and a higher risk of falls [2, 17, 18]. Moreover, the peripheral nervous system regulates gait through somatic and autonomic functions, coordinating eccentric and concentric muscle contractions while processing sensory input from the plantar surface [3]. These sensory deficits in individuals with DPN result in cautious walking strategies, such as slower speeds, reduced cadence, and increased variability in step length and velocity [19]. The relationship between sway, abnormal gait, and increased falls has been confirmed [18].

The risk of falls in individuals with DPN is multifactorial, influenced by cognitive impairment, depression, orthostatic hypotension, and hypoglycemic episodes. Additionally, dysfunction in the visual, vestibular, somatosensory, and musculoskeletal systems further compromises postural stability. Although fall assessment methods vary across studies, there is consistent evidence of elevated fall risk among individuals with DPN [20]. Recent studies suggest that vibrating insoles may acutely improve dynamic balance and gait performance, offering a novel strategy to enhance mobility and mitigate fall risk. Reported benefits include enhanced balance, increased gait speed, therapeutic potential, and improved sensory feedback [21].

Balance and gait impairments are common in individuals with DPN. To capture functional deficits not typically identified through standard neurological examinations, this study was complemented by detailed assessments of balance and gait, including static postural sway measurements and gait analysis of spatiotemporal parameters and joint range of motion (ROM) assessment during walking. These functional evaluations were conducted in conjunction with a validated clinical neuropathy questionnaire and assessments of both small and large nerve fiber function. The objective was to examine the feasibility and diagnostic value of balance and gait assessments as screening tools for DPN. A clearer understanding of balance and gait impairments in participants with DPN may inform the design of targeted rehabilitation strategies to mitigate fall risk and enhance functional outcomes in this population.

### Participants and study design

Participants for this study were recruited from residents of a nearby daycare center affiliated with our hospital and their family members from the surrounding community to assess the impact of DPN on balance and gait. All participants were newly recruited and had no prior registration with our hospital. Participants with moderate to severe disabilities due to central or peripheral nerve disorders or cognitive impairments preventing them from following instructions were excluded.

The sample size (n) was calculated using G*Power software (version 3.1.9.2), developed by the University of Düsseldorf, Germany [22]. The significance level was set at α = 0.05, with a two-tailed statistical test. Considering a correlation of 0.5 between repeated measures, the statistical power was set at 1-β = 0.8. This study included three groups: the DM with DPN group, the DM without DPN group, and the healthy participant group. The required sample size was determined to be 159 using a fixed-effects model in one-way analysis of variance. A total of 146 participants were enrolled. All participants first underwent peripheral nerve function evaluations, followed by assessments of balance and gait.

In this community-based study, diabetes mellitus was diagnosed either through medical history or a random finger stick blood glucose reading of over 200 mg/dL (11.1 mmol/L). DSPN is diagnosed in patients with diabetes who have typical symptoms and symmetrical distal sensory loss or who are asymptomatic but have typical signs [10, 23]. The Toronto Clinical Neuropathy Score (TCNS) was assessed, with a minimum score of 5 indicating a diagnosis of DPN [24]. Informed consent was obtained from all participants, and the study was approved by the Institutional Review Board of the hospital (Approval No. 202300962B0).

### Clinical assessment

Baseline evaluations included comprehensive neurological examinations using the TCNS, a validated tool for diagnosing and assessing the severity of DPN [25]. A TCNS score of 5–8 is considered indicative of mild DPN, 9–11 as moderate DPN, and ≥ 12 as severe DPN [24]. Baseline demographic data, including height (cm), weight (kg), body mass index (BMI), waist circumference (cm), systolic and diastolic blood pressure (mmHg), and the presence of underlying medical conditions, were obtained through patient interviews and physical examinations.

### Large and small nerve Fiber measurements

Large nerve fiber function was assessed using NCS with Nicolet Viking machines (Madison, Wisconsin). Measurements included sensory nerve action potential (SANP) and sensory nerve conduction velocity (SNCV) of the sural nerves, conducted according to our previously published protocol [26]. Small fiber function was evaluated using the Sudoscan device [27], which measures electrochemical skin conductance (ESC) by applying a low-voltage current (< 4 V) to stimulate small fibers innervating the sweat glands. ESC measurements were taken from hands and feet.

### Balance and gait assessments

Balance and gait analyses were conducted in participants with sufficient lower extremity strength for antigravity movement and independent walking. The assessment protocol included static postural sway measurements and gait analysis of spatiotemporal parameters and joint ROM assessment during walking.

### Postural sway measurements

**Fig. 1:**
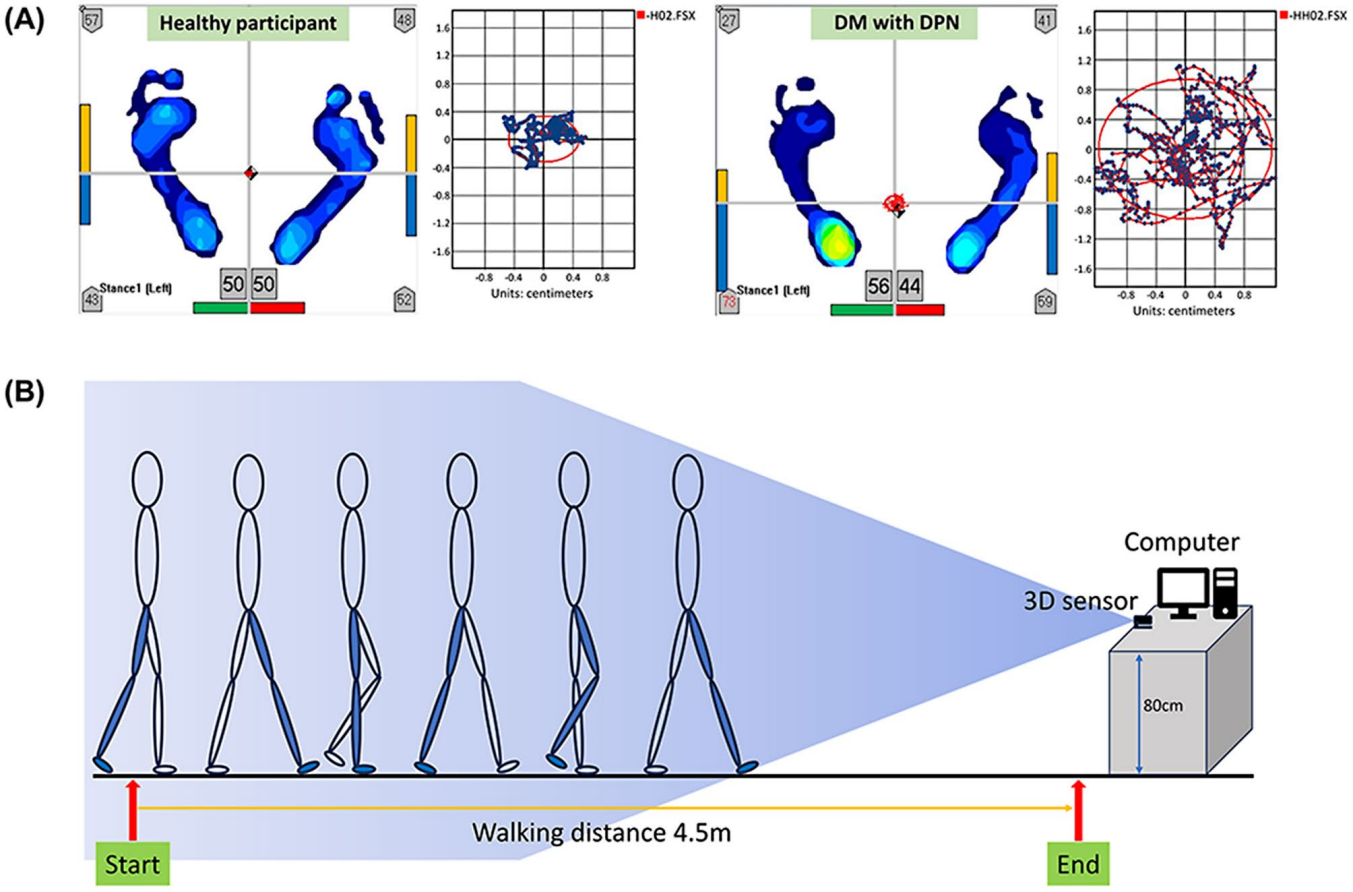
Illustrates balance and gait analysis. Panel **A** presents CoP trajectories in the horizontal plane during standing postural sway in a healthy participant and a participant with DPN. Panel **B** shows the walkway used for gait and kinematic analysis

Postural sway, defined as the involuntary oscillatory movement of the CoP within the base of support to maintain balance, was assessed using the TekScan MatScan pressure mat (Model 3150, TekScan Inc., South Boston, USA). Postural sway was recorded with the following data acquisition settings: 30 s, 900 movie frames, a sampling frequency of 30 Hz, a frame period of 0.033 s, and a noise threshold set at 3. This evaluation quantified key sway parameters, including area (cm²), length (cm), velocity (cm/s), and excursion in both the AP and ML directions, providing essential insights into postural stability and balance control (Fig. 1A). Data were analyzed using the Sway Analysis Module (SAM™) software. After calibration, sway was recorded three times for each participant during a 30-second trial while standing barefoot with feet together, arms at the sides, and eyes open. The average of the three trials was used for analysis.

### Gait and kinematics analysis

Gait and kinematic data were captured using Kinect V2 sensors, which tracked 25 key joint points at a 30 Hz sampling rate. The GaitBEST algorithm (LONGGOOD MEDITECH LTD., Taipei, Taiwan), running on a Windows 10 platform with an i5 or higher CPU, was used for data analysis as described by Lai et al. [28]. The system automatically captures joint positions to compute key temporal markers, displacements, and spatiotemporal and kinematic parameters. The gait analysis software applies a calibration error correction method based on a theoretically sound algorithm, comparing measured data with normative values to exclude outliers. Outliers are identified through an initial comparison with normative data, followed by recalculating the mean and standard deviation; data points exceeding 1.5 standard deviations are discarded. This process ensures data stability, and normal distribution, and minimizes measurement errors.

Participants initiated gait with the right leg. The sensor camera was positioned on an 80 cm-high platform, with an unobstructed area of 2.5 m wide and 4.5 m long in front. The walkway was aligned perpendicular to the sensor, and participants walked a distance of 4.5 m (Fig. 1**(B)).** During the initial acceleration phase, the system automatically excludes data corresponding to the first 0.3 m, and during the deceleration phase, it omits the final 1.0 m of data to minimize the influence of transitional gait dynamics on the analysis.

### Statistical analysis

Data were presented as mean ± SD. Four statistical analyses were performed. First, trends among the three groups (healthy controls, diabetes with and without DPN were analyzed using analysis of variance (ANOVA) with Welch’s correction, followed by the Games–Howell post hoc test. Second, significant gait and balance variables from ANOVA were included in a stepwise logistic regression model, adjusted for age, diabetes duration, and height, to predict DPN presence. The model was calibrated by the Hosmer-Lemeshow goodness-of-fit test. The test results, under a χ2 distribution, provide a P -value in which higher values (*P* > 0.05) indicate nonsignificant differences between observed and predicted DPN. Due to notable correlations among explanatory variables from postural sway and spatiotemporal parameters, independent risk factors were identified using the least absolute shrinkage and selection operator (LASSO) regression method. Third, the AUC, sensitivity, specificity, and Youden’s index were determined for each significant gait and balance parameter from the final logistic regression model. Finally, correlation analyses were conducted between significant variables, clinical scores, sural nerve measurements, and ESC. Statistical analyses were conducted using IBM SPSS Statistics v26 (IBM, Redmond, WA, USA).

## Results

### Baseline patient characteristics

Among the 146 participants, 35 had diabetes, with 22 having DPN and 13 without DPN. The remaining 111 participants were healthy controls. The mean duration of diabetes was 11.14 ± 7.77 years in those with DPN and 10.08 ± 9.65 years in those without DPN. Baseline characteristics and comorbidities, including hypertension as the most prevalent, followed by hyperlipidemia, are detailed in Table 1. TCNS progressively increased across groups, with the lowest values observed in healthy participants (1.78 ± 0.86), followed by those with diabetes without DPN (1.92 ± 1.75), and the highest in participants with DPN (8.95 ± 2.48). This difference was statistically significant (*p* < 0.0001). In contrast, sural SNAP, SNCV, and ESC values were highest in healthy participants, lower in those with diabetes without DPN, and lowest in those with DPN. Specifically, sural SNAP values were 12.69 ± 5.12 µV in healthy participants, 8.92 ± 3.67 µV in participants with diabetes without DPN, and 7.06 ± 4.55 µV in those with DPN (*p* < 0.0001). Corresponding SNCV values were 45.92 ± 6.31 m/s, 39.15 ± 13.27 m/s, and 34.55 ± 17.68 m/s, respectively (*p* = 0.01), while foot ESC values were 65.10 ± 17.52 µS, 60.15 ± 19.27 µS, and 48.09 ± 19.95 µS, respectively (*p* = 0.005).

**Table 1:** Clinical characteristics and demographics of the study population

	Healthy participants (*n* = 111) (%)	DM without DPN (*n* = 13) (%)	DM with DPN (*n* = 22) (%)	*P*-value
**Baseline characteristics**				
Age (year)	70.86 ± 10.88	71.15 ± 6.11	72.95 ± 8.88	0.80
Sex (male/ female)	39/72	6/7	11/11	0.35
Diabetes duration (year)	--	10.08 ± 9.65	11.14 ± 7.77	0.74
Height (cm)	160.40 ± 8.17	159.38 ± 7.94	161.41 ± 8.52	0.78
Body mass index	24.15 ± 3.62	25.76 ± 3.02	26.90 ± 5.25	0.04
Waist circumstance (cm)	82.83 ± 9.16	91.35 ± 10.20	93.75 ± 11.98	< 0.0001^α^
Systolic blood pressure (mmHg)	127.79 ± 17.27	134.15 ± 18.0	134.73 ± 13.08	0.11
Diastolic blood pressure (mmHg)	78.71 ± 9.90	71.92 ± 14.59	72.64 ± 12.20	0.06
Baseline underlying disease				
Hypertension (%)	25 (22.5)	11 (84.6)	17 (77.3)	< 0.0001
Hyperlipidemia (%)	21 (18.9)	12 (92.3)	12 (54.5)	< 0.0001
Smoking (%)	14 (12.6)	1 (7.7)	2 (9.0)	0.80
Coronary heart disease (%)	3 (2.7)	1 (7.7)	0	0.40
Ischemic stroke (%)	0	1 (7.7)	3 (13.6)	0.001
**TCNS**	1.78 ± 0.86	1.92 ± 1.75	8.95 ± 2.48	< 0.0001^β^
**NCS**				
Sural SNAP, left (µV)	12.69 ± 5.12	8.92 ± 3.67	7.06 ± 4.55	< 0.0001^γ^
Sural SNCV	45.92 ± 6.31	39.15 ± 13.27	34.55 ± 17.68	0.01^η^
**Sudoscan**				
Hand ESC, µS	63.94 ± 18.18	54.77 ± 19.65	45.68 ± 21.03	0.003^θ^
Foot ESC, µS	65.10 ± 17.52	60.15 ± 19.27	48.09 ± 19.95	0.005^ι^

Data are presented as means ± standard deviations or n (%). Abbreviations: n, number of cases; HbA1C = glycohemoglobin; TCNS, Toronto Clinical Neuropathy Score; SNAP = sensory nerve action potential; SNCV = sensory nerve conduction velocity; ESC = electrochemical skin conductance

α = Healthy participants vs. DM without DPN, *p* = 0.03; Healthy participants vs. DM with DPN, *p* = 0.001

β = Healthy participants vs. DM with DPN, *p* < 0.0001; DM without DPN vs. DM with DPN, *p* < 0.0001

γ = Healthy participants vs. DM without DPN, *p* = 0.01; Healthy participants vs. DM with DPN, *p* < 0.0001

η = Healthy participants vs. DM with DPN, *p* = 0.2

θ = Healthy participants vs. DM with DPN, *p* = 0.002

ι = Healthy participants vs. DM with DPN, *p* < 0.0001

### Balance and gait assessments of the study population

Table 2 provides a comparative analysis of balance and gait assessments across three groups: healthy participants, participants with diabetes without DPN, and those with DPN. Postural sway velocity (cm/s) was significantly lower in healthy participants (0.73 ± 0.33) compared to participants with diabetes without DPN (0.95 ± 0.53) and those with DPN (1.20 ± 0.53; *p* = 0.002). Similarly, total path length (cm) increased across groups, with values of 21.88 ± 9.75 cm, 28.57 ± 15.99 cm, and 35.98 ± 15.93 cm, respectively (*p* = 0.002). ML path length (Length x) also increased progressively: 1.26 ± 0.55 cm in healthy participants, 1.45 ± 0.82 cm in those with diabetes without DPN, and 1.84 ± 0.94 cm in those with DPN (*p* = 0.03).

**Table 2:** Balance and gait assessment of the study population

		Healthy participants (*n* = 111) (%)	DM without DPN (*n* = 13) (%)	DM with DPN (*n* = 22) (%)	*P*-value
**Standing postural sway**					
Area (cm^2^)		1.19 ± 0.99	1.55 ± 1.02	2.01 ± 1.63	0.09
Velocity (cm/s)		0.73 ± 0.33	0.95 ± 0.53	1.20 ± 0.53	0.002^α^
Length (cm)		21.88 ± 9.75	28.57 ± 15.99	35.98 ± 15.93	0.002^β^
Length y (cm)		1.83 ± 0.75	2.10 ± 1.10	2.26 ± 0.82	0.08
Length x (cm)		1.26 ± 0.55	1.45 ± 0.82	1.84 ± 0.94	0.03^γ^
**Spatiotemporal parameters**					
Cadence(steps/s)		1.86 ± 0.19	1.78 ± 0.15	1.80 ± 0.18	0.09
Speed (m/s)		1.09 ± 0.20	1.0 ± 0.13	0.91 ± 0.24	0.005^η^
Stride length(m)		1.16 ± 0.18	1.13 ± 0.15	1.01 ± 0.24	0.04^θ^
Step length variability (CV)		13.8 ± 6.08	11.96 ± 3.67	17.01 ± 8.92	0.07
Stride time(s)		1.08 ± 0.11	1.13 ± 0.09	1.12 ± 0.12	0.14
Step time variability (CV)		13.16 ± 4.86	12.43 ± 4.14	13.93 ± 4.98	0.64
**Kinematics parameters**					
Shoulder Flex/Ext ROM (°)		24.37 ± 9.47	23.16 ± 6.52	24.73 ± 9.01	0.81
Elbow Flex/Ext ROM (°)		16.63 ± 7.12	14.49 ± 7.45	14.82 ± 6.40	0.39
Trunk bending ROM (°)		3.64 ± 1.50	4.05 ± 1.37	3.15 ± 0.97	0.19
Trunk sway ROM (°)		3.04 ± 1.29	3.42 ± 1.60	3.36 ± 1.75	0.47
Trunk rotation ROM (°)		10.92 ± 3.76	10.61 ± 2.79	10.77 ± 3.87	0.95
Hip Flex/Ext ROM (°)		60.53 ± 8.29	57.94 ± 5.37	53.91 ± 7.39	0.05
Knee Flex/Ext ROM (°)		72.60 ± 7.18	70.45 ± 4.74	70.21 ± 6.57	0.14

Abbreviations: CV = coefficient of variation; ROM (degree) = range of motion; degree = 。, Area (cm2), length (cm), and velocity (cm/s) traveled by the excursion (mm) of the center of pressure (CoP), Length y indicates path length in the anterior-posterior direction. Length x indicates path length in the medio-lateral direction; z = Track velocity (m/s) in the segments in the anteroposterior direction; x = Track velocity (m/s) in the segments and in the mediolateral direction; CV = coefficient of variation; ROM (degree) = range of motion; degree = 。

Bonferroni’s multiple comparison for post-hoc test:

α = Healthy participants vs. DM with DPN, *p* = 0.001; β = Healthy participants vs. DM with DPN, *p* = 0.001; γ = Healthy participants vs. DM with DPN, *p* = 0.025; η = Healthy participants vs. DM with DPN, *p* = 0.008; θ = Healthy participants vs. DM with DPN, *p* = 0.03

Regarding spatiotemporal parameters, gait speed, and stride length were highest in healthy participants, intermediate in participants with diabetes without DPN, and lowest in those with DPN (*p* = 0.001 and *p* = 0.003, respectively). Specifically, gait speed (m/s) was 1.09 ± 0.20 in healthy participants, 1.00 ± 0.13 in those without DPN, and 0.91 ± 0.24 in those with DPN (*p* = 0.002). Stride length (m) followed a similar pattern, with values of 1.16 ± 0.18, 1.13 ± 0.15, and 1.01 ± 0.24, respectively (*p* = 0.04). Hip flexion/extension ROM was highest in healthy participants (60.53 ± 8.29), followed by those with diabetes but without DPN (57.94 ± 5.37), and lowest in those with DPN (53.91 ± 7.39) (*p* = 0.05). Additional balance and gait parameters are detailed in Table 2.

ANOVA with Welch’s correction, followed by the Games–Howell post hoc test, revealed significant differences only between healthy participants and participants with diabetes and DPN. These differences were observed in postural sway velocity (*p* = 0.002), total path length (*p* = 0.002), length x (*p* = 0.03), walking speed (*p* = 0.005), stride length (*p* = 0.04) and hip flexion/extension ROM (*p* = 0.05). No statistically significant differences were found between healthy participants and participants with diabetes without DPN or between participants with diabetes with and without DPN.

### Balance and gait parameters as predictors of DPN: a logistic regression model

Significant balance and gait variables identified through ANOVA in Table 2—including postural sway velocity (cm/s), total path length (cm), ML path length (Length x, cm), gait speed (m/s), stride length (m) and hip flexion/extension ROM—were incorporated into stepwise logistic regression models, along with age, diabetes duration, and height, to evaluate their predictive value for the presence of DPN. The analysis identified diabetes duration and postural sway velocity (cm/s) as the only significant predictors of DPN presence (Table 3). Moreover, to address multicollinearity among predictors, LASSO regression analysis was employed. This approach identified two variables with non-zero coefficients—diabetes duration and postural sway velocity (cm/s)—as independent predictors of DPN. The stepwise logistic regression model was calibrated by the Hosmer-Lemeshow goodness-of-fit test, indicating no significant discrepancy between the observed and predicted model (Chi-square = 13.59, df = 8, *P* = 0.093). To further evaluate the predictive value of postural sway velocity, ROC curve analysis was conducted. The ROC results showed a sensitivity of 68.2%, a specificity of 85.5%, and an AUC of 0.76 (95% CI: 0.63–0.88, *p* < 0.0001), with a cut-off value of 0.98 cm/s.

**Table 3:** Balance and gait parameters on the predictor of presence of diabetic peripheral neuropathy: A logistic regression model

Significant univariable^†^	β	S.E.	OR	95% CI	*P*-value
Constant	-3.74	0.66	0.024		< 0.0001
Diabetes duration	0.18	0.04	1.19	1.10–1.30	< 0.0001
Postural sway Velocity (cm/s)	1.28	0.57	3.58	1.17–10.96	0.025

**†**: Only list those significant variables in On-way ANOVA were enrolled in the stepwise logistic regression model

β = Regression coefficients, S.E.=standard error, OR = Odds ratio, CI = confidence intervals

### Correlation analysis between postural sway velocity and TCNS, NCS, and ESC

**Fig. 2:**
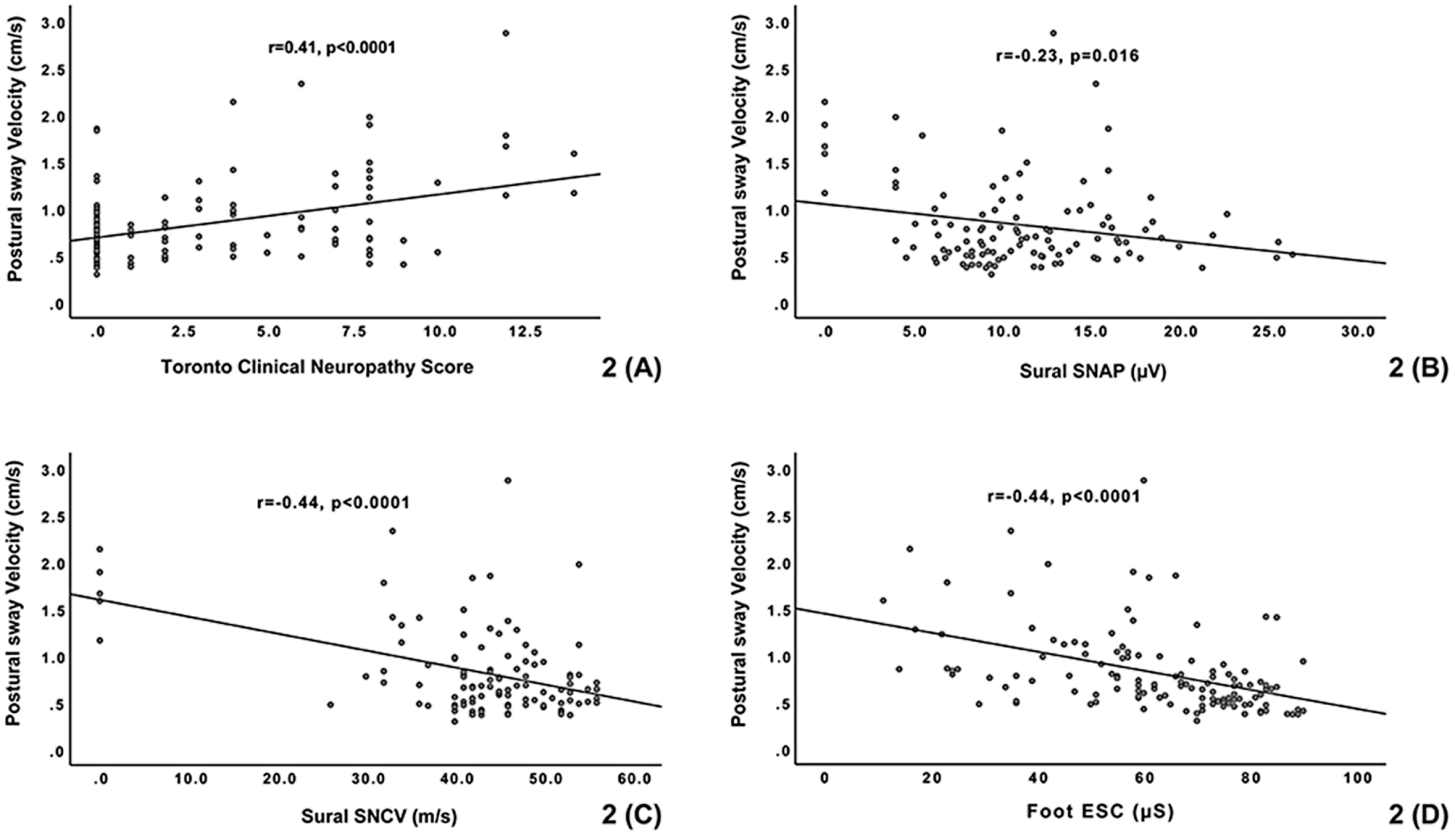
Relationship between postural sway velocity and (**A**) Toronto Clinical Neuropathy Score (TCNS), (**B**) Sural sensory nerve action potential (SNAP), (**C**) Sural sensory nerve conduction velocity (SNCV), and (**D**) foot electrochemical skin conductance (ESC) in participants

A correlation analysis was performed to examine the relationship between postural sway velocity and the TCNS, NCS, and ESC. Significant correlations were found between postural sway velocity and TCNS (*r* = 0.41, *P* < 0.0001), sural SNAP (*r* = -0.23, *P* = 0.016), sural SNCV (*r* = -0.44, *P* < 0.0001), hand ESC (*r* = -0.38, *P* < 0.0001), and foot ESC (*r* = -0.44, *P* < 0.0001), as summarized in Fig. 2.

## Discussion

### Major findings of the study

Our study provides compelling evidence of significant balance and gait impairments in participants with DPN. Participants demonstrated increased postural sway velocity, total path length and length in ML direction (length x), reduced walking speed and stride length, and decreased hip flexion/extension ROM. Additionally, postural sway velocity was significantly correlated with higher TCNS, reduced sural SNCV, and SNAP, as well as lower ESC values. These findings suggest postural sway velocity could serve as an early marker for DPN screening, supporting its role in clinical assessments and early interventions.

### Association between large and small fiber function and balance and gait parameters

Among NCS parameters, sural SNAP amplitude is essential for diagnosing DSPN, reflecting axonal count [29], while sural SNCV strongly correlates with DSPN severity [30]. Sudoscan™ assesses small fiber neuropathy and autonomic dysfunction [12, 31], with foot ESC correlating with intraepidermal and sweat gland nerve fiber density in the distal leg [32]. In addition, studies employing small fiber techniques have identified subclinical abnormalities in small unmyelinated Aδ and C fibers, which are critical for pain and temperature perception [33, 34]. In our study, we found that balance and gait parameters varied significantly across clinical classifications (healthy, diabetes without DPN, and diabetes with DPN). Moreover, postural sway velocity demonstrated a statistically significant correlation with both sural nerve conduction measures and ESC, suggesting that while large fiber dysfunction remains the primary contributor to impaired proprioception and motor control, small fiber dysfunction may also play a complementary role in sensorimotor instability.

### Balance and gait impairments in diabetic peripheral neuropathy

Balance is crucial for postural control and fall prevention [35]. Participants with DPN experience significant challenges in postural control due to impaired sensory feedback, resulting in gait and balance impairments that substantially elevate fall risk [36]. As DPN progresses, balance deteriorates further, evidenced by increased postural sway [17, 37–39]. A study investigating the impact of postural stability on DPN found a significant correlation between neuropathy severity and postural stability [38]. As neuropathy severity increased, postural stability decreased, with patients exhibiting larger sway ranges, faster sway speeds, and greater sway dispersion compared to controls. These findings indicate that balance control is closely linked to DPN severity, highlighting an elevated fall risk, particularly during complex daily tasks, in participants with more severe symptoms [38]. Our findings are consistent with this, showing that participants with DPN have increased postural sway velocity, and longer postural sway trace length [17, 37, 38]. Additionally, postural sway velocity emerged as the most reliable surrogate marker for balance and gait impairment in participants with DPN in our study. A study by Almurdhi et al. found that participants with impaired glucose tolerance, but not type 2 diabetes, exhibited significantly increased ML sway during walking, suggesting gait changes may start in the pre-diabetes stage [40]. Our study found that participants with DPN showed significantly greater total postural sway, particularly in the ML direction, compared to healthy controls. This increased sway may result from reduced use of ankle movements (ankle strategy), especially when visual input is limited, leading to poorer balance control [38]. Since the hip joints play a key role in ML stability—through the action of the hip abductor and adductor muscles—individuals with DPN may rely more on hip movements (hip strategy) to compensate for impaired ankle function [41, 42]. These results are consistent with previous studies reporting greater ML instability in individuals with DPN, even when visual input is available [2, 43].

Participants with DPN exhibit reduced ROM at key joints, particularly decreased dorsiflexion and plantar flexion at the ankle, and reduced knee flexion and extension compared to non-diabetic participants [44, 45]. These ankle ROM alterations may be linked to changes in plantar pressure [46]. Kinematic alterations in individuals with DPN, including reduced flexion and extension at the knee and hip joints, are associated with decreased walking speed, shortened stride length, and increased gait variability [3]. Studies have shown that individuals with more severe DPN-related loss of plantar cutaneous sensation tend to exhibit a slower preferred walking speed [47, 48]. Our study supported previous findings by showing that participants with DPN had significantly slower walking speeds and shorter stride lengths compared to healthy individuals. Although step length variability was higher in the DPN group, the difference was not statistically significant. These impairments compromise stability, increasing fall risk. Hip muscle weakness and reduced mobility further contribute to gait dysfunction, often requiring compensatory strategies. Reduced hip ROM may compensate for restricted ankle movement, further affecting gait dynamics and fall risk. Early intervention is crucial to address these challenges.

### Rehabilitation strategies targeting diabetic peripheral neuropathy

This systematic review and meta-analysis critically evaluate the effectiveness of physical therapy interventions, including manual therapy and stretching exercises, in improving ankle and hallux ROM, reducing peak plantar pressures on the plantar surface of the foot, and minimizing postural sway in participants with diabetes [49]. These interventions hold the potential to enhance mobility, stability, and overall quality of life for participants living with diabetes [49]. The other systematic review suggests that balance-focused rehabilitation interventions, such as Tai Chi and yoga, can improve postural control in participants with DPN at risk of falling [50]. However, the impact on fall reduction remains inconclusive due to heterogeneous methodologies and limited long-term follow-up. In our view, balance-focused rehabilitation strategies such as Tai Chi and yoga should be integrated into early clinical interventions to actively enhance postural control in participants with DPN at risk of falling [50], whereas vibrating insoles may be more suitable for passive fall prevention in those with advanced DPN due to safety considerations [21]. Further research is needed to establish the link between balance improvements and fall incidence to enhance the effectiveness of targeted interventions.

### Strengths and limitations of the study

Participants with DPN demonstrate greater postural sway velocity and total path length, slower gait speed, shorter stride length, and reduced hip ROM. Postural sway velocity, identified via LASSO regression as an independent predictor, may serve as a promising early biomarker for DPN screening. These findings support the value of balance and gait assessments in detecting functional impairments that may not be captured by standard neurological evaluations. However, the study has several limitations. First, the relatively small number of participants with diabetes and variability in participants’ physical characteristics (e.g., baseline fitness) may limit the accuracy and generalizability of the findings. Second, the cross-sectional design precludes causal inference regarding the relationship between gait and balance impairments and the progression of DPN. Third, generalizability may be restricted due to recruitment from a single community setting. In this study, participants were mainly recruited through the daycare center adjacent to our hospital and included members of the center and the local community, without prior registration at our hospital. To support community-based implementation, we temporarily relocated specialized equipment (e.g., NCS, Sudoscan, gait, and balance systems) to the daycare center to create a controlled yet accessible testing environment. While this setup does not reflect the logistical realities of most community settings, it served as a prototype model to explore feasibility. In future applications, simpler and more scalable technologies—such as wearable inertial sensors or smartphone-based balance tools—may replace the research-grade systems used here. Thus, while our current approach involves specialized tools, the conceptual framework is aimed at eventual adaptation into more accessible, field-ready screening methods. Additional studies using lower-cost and portable technologies are needed to confirm its utility in true low-resource settings. Fourth, our study aims to develop a community-based screening service for DPN using gait and balance assessments. NCS was limited to assessing the sural nerves. The inclusion of additional nerve assessments, such as the tibial and peroneal nerves, could have enhanced diagnostic sensitivity by identifying cases where only one sural nerve was affected. Although Kinect V2 is a cost-effective and accessible tool for assessing spatiotemporal parameters and joint ROM, its accuracy is lower than that of gold-standard methods such as motion capture systems. Kinect V2 demonstrated moderate to excellent accuracy in landmark tracking, influenced by movement dimension, landmark location, and task, supporting its potential as a reliable clinical tool [51]. Despite limited reliability in ankle detection [52], clinical studies confirm the validity of Kinect-based kinematic measurements [53]. A more definitive understanding of how balance and gait impair DPN progression requires prospective, longitudinal designs rather than cross-sectional snapshots. Recruiting participants at different stages of DPN (from prediabetes to clinically overt DPN) may be an approach, or incorporating interventions (e.g., balance training or rehabilitation programs) into randomized controlled trials could determine whether improved gait mechanics alter the natural course of DPN or primarily reduce functional decline.

## Conclusion

Balance and gait impairments are commonly observed in participants with DPN, indicating their potential utility as screening measures. Among these, postural sway velocity may serve as a promising early surrogate biomarker for DPN detection. This study supports its utility in early detection and emphasizes the importance of addressing balance deficits to guide targeted fall prevention interventions.

## Data Availability

No datasets were generated or analysed during the current study.

## Declarations

### Animal studies

N/A.

## Abbreviations

AUC: Areas under the receiver operating characteristic curves
AP: Anteroposterior
DPN: Diabetic peripheral neuropathy
DSPN: Diabetic sensorimotor polyneuropathy
ESC: Electrochemical skin conductance
ML: Mediolateral
NCS: Nerve conduction studies (NCS)
ROM: Range of motion
SNCV: Sensory nerve conduction velocity
SNAP: Sensory nerve action potential
TCNS: Toronto clinical neuropathy score
